# Beneficial effects of arthrocen on neuroinflammation and behavior like depression in stroke in a murine model

**DOI:** 10.1002/fsn3.3083

**Published:** 2022-10-05

**Authors:** Ramin Goudarzi, Golnaz Zamanian, Zahra Seyyedian, Partow Mirzaee Saffari, Ahmad Reza Dehpour, Alireza Partoazar

**Affiliations:** ^1^ Division of Research and Development, Pharmin USA LLC San Jose California USA; ^2^ Department of Pharmacology, School of Medicine Tehran University of Medical Sciences Tehran Iran; ^3^ Experimental Medicine Research Center Tehran University of Medical Sciences Tehran Iran

**Keywords:** animal model, arthrocen, avocado/soybean unsaponifiables, bioactive natural, brain ischemia, herbal, ROCEN

## Abstract

Stroke is a considerable reason for death, disability, socioeconomic loss, and depression in the world. Notably, many attempts to the reduction of the complications of poststroke injuries like depression have failed so far. In this study, we aimed to evaluate the anti‐inflammatory effect of arthrocen, avocado/soybean unsaponifiables (ASU), in the poststroke injuries like depression improvement in a mice model. We examined the antidepressant‐like effect of arthrocen using the forced swimming test and tail suspension test in mice subjected to stroke. Furthermore, immunohistochemistry of proinflammatory cytokines, IL‐10 and TNF‐α, and neural cell count were performed in the ischemic brain hippocampus of mice. Oral arthrocen reduced significantly (*p* < .001) the immobility time in the forced swimming test and tail suspension test in the stroke animals. Also, immunohistochemistry analysis of the hippocampus indicated significantly (*p* < .01) the reduction of IL‐10 and TNF‐α cytokines production. Nissl staining showed a significant (*p* < .0001) increase in the number of viable neurons in stroke mice receiving arthrocen. In conclusion, our data revealed the antidepressant activity of arthrocen in the stroke mice which may be the result of its anti‐inflammatory and neuroprotective role.

## Graphical abstract

Following oral administration of arthrocen supplement in post‐stroke depression model, the study recommended a novel protective effect by arthrocen on neuroinflammation due to stroke. It may play an antidepressant role through anti‐inflammatory as well as improved activity and mobility in mice‐induced stroke.

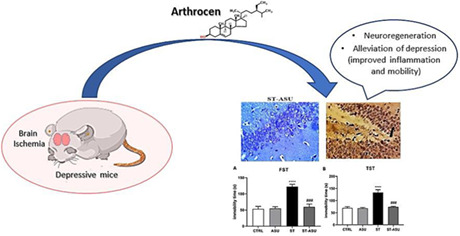

## 1 INTRODUCTION

Poststroke depression (PSD) is a well‐known complicated disease that affects approximately one‐third of the survivors of stroke patients (Towfighi et al., 2017). The progression of depression in stroke (ST) is clinically important because it can be associated with morbidity or mortality (Bartoli et al., 2013). Symptoms such as negative mood against interest in activities, changes in appetite, and sleep decreased, are major symptoms identified in depressive patients (Mitchell et al., 2017; Sher et al., 2013). It is believed that PSD can be even a factor in ST recurrence among ischemic patients (Yuan et al., 2012).

The positive effects of antidepressants on the remission of depressive symptoms have been observed in patients with PSD (Kraus et al., 2019). The delayed onset of action of the antidepressants, adverse drug effects, and noncompliance in patients using current drugs is a major problem in this disorder (Paolucci, 2008). Therefore, the development of effective therapeutics and preventive approaches will be desirable to reduce the burden and morbidity associated with PSD (Bartoli et al., 2013).

It is determined that the progression of neurological disorders of ST‐like depression is a result of the acceleration of inflammation in the tissue brain (Hu et al., 2021) and evidence recommends the control of the inflammatory factors as a promising approach against PSD (Drieu et al., 2018; Fang et al., 2019). In several studies, inflammatory cytokines of TNF‐α, IL‐1β, IL‐6, IL‐10, etc. were reported as a biomarker involved in PSD disorder illnesses (Audet & Anisman, 2013; Ma et al., 2016). Other studies also showed that inflammatory factors such as IL1β, TNF‐α, and Tlr‐4 are involved in the development of depressive disorders which can be hindered by bioactive natural compounds in animal models (Arabi et al., 2021; Lorigooini et al., 2021).

Arthrocen, avocado/soybean unsaponifiables (ASU), compound is an oily extract containing phytosterols, lipophilic vitamins, and terpenoids, classified as a dietary supplement in the US. Previous studies have shown that ASU compound plays a key role in the treatment of osteoarthritis (OA), reducing pain and inflammation (Goudarzi, Nasab, et al., 2020; Taylor et al., 2017), as well as reducing the expression of inflammatory cytokines and prostaglandin E2 and inhibition of COX‐2 (Au et al., 2007). Recent studies have also shown that ASU has anticonvulsant (Goudarzi, Zamanian, et al., 2020) and anti‐ischemic effects (Yaman et al., 2007) in animal models.

Regarding potentiation of ASU in the suppression of inflammation as well as neuroprotection in brain disorders, it is recommended to restudy the antidepressive activity of ASU in the post‐ST state. ST due to common carotid artery obstruction is proposed to show signs of depression in animal models, which can be similar to the real state of PSD in the clinic. For example, experimental PSD or cognitive deficits with hippocampal inflammation (Partoazar et al., 2021) or atrophy (Yoshizaki et al., 2008), respectively, have been described by inducing the unilateral common carotid artery occlusion (UCCAO) in animal models. Therefore, we aimed to evaluate the potential effect of anti‐inflammatory ASU on the improvement of PSD outcomes through inhibition of neuroinflammation in ST mice.

## 2 MATERIALS AND METHODS

### 2.1 Animals

In this experiment, we used a total of 24 male NMRI mice weighing 20–25 g (Pasteur Institute, Tehran). Animals were housed with free access to food and water and were kept under standard laboratory conditions (temperature 21–23°C and 12‐h light/dark cycle). All behavioral experiments were conducted to time between 10:00 and 16:00 with normal room light. The experimental protocols were approved by the Ethics Committee of Tehran University of Medical Sciences (No. 1400.1496) in agreement with the standards for the care and use of laboratory animals.

### 2.2 Experimental design

In this experiment, the right unilateral common carotid artery occlusion (rUCCAO) was operated on mice‐inducing ST. The four experimental groups were provided randomly from six mice and divided into the following trials:

1. CTRL group: 6‐day saline administration, no operation, 72‐h saline administration;
2. ST group: 6‐day saline administration, rUCCAO surgery, 72‐h saline administration;
3. ASU group: 6‐day ASU administration, no operation, 72‐h ASU administration;
4. ST‐ASU group: 6‐day ASU administration, rUCCAO surgery, 72‐h ASU administration.

Arthrocen manufactured by Pharmin USA, LLC was dissolved in saline and administered orally to the ST‐ASU and ASU groups by gavage at the dose of 50 mg/kg/d for 9 days. On the ninth day, experiments were evaluated by the behavioral tests, forced swimming test (FST), and tail suspension test (TST). Finally, animals were euthanized to separate whole‐brain samples and placed in buffered formalin (10%) for the histopathology examination.

### 2.3 Ischemic stroke procedure

In our study, we subjected UCCAO in mice through the permanently double ligations of right common carotid artery to induce the ischemic ST model (Gooshe et al., 2015). Before surgery, animals were under ketamine (50 mg/kg, IP) and xylazine (10 mg/kg, IP) anesthetizers. When an absent reflex by the test was confirmed in the animal, subjected tissues were sterilized and an anterior midline incision was provided. The right common carotid artery was isolated from connected tissues and the vagus nerve. Then, the isolated artery was ligated in two sites and cut between the cords. Finally, the midline incision in the mouse was sterilized and then sutured with the silk 4–0 suture.

### 2.4 Depression evaluations

#### 2.4.1 Forced swimming test (FST)

The FST test according to previous studies as a standard rodent test was used to assess behavioral despair in mice for screening antidepressant activity of drugs (Ostadhadi et al., 2018). Seventy‐two hours after ST induction, mice in each group were tested under FST. Animals were placed in an open cylinder‐shaped flask (diameter 10 cm, height 25 cm) containing 19 cm of water at 24 ± 1°C. Each mouse was regarded as immobile when stopped struggling and floated motionless on the water, and making only the movements necessary for keeping its head above water. The behaviors were distinguished in a 6‐min period while only the duration of immobility within the last 4 min was recorded with a digital stopwatch.

#### 2.4.2 Tail suspension test (TST)

Stroke mice in each group were tested for TST at 72 h after surgery. According to the experimental method (Partoazar et al., 2021), animal was suspended on the tip of its tail using scotch tape for attachment to the edge of a rod that was 50 cm above the tabletop. Tail climbing was forbidden by a small cylindrical holder before the suspension of mice. Animal in an immobile state was indicated by a completely hanging down position and when it remained motionless.

### 2.5 Histopathology and immunohistochemistry analysis

After behavior tests, animals were sacrificed and their brains separated, fixed, and cut into coronal sections of 4‐μm thickness. Two coronal sections (300 μm apart) through the hippocampus were processed for staining with Cresyl violet 0.1% (Nissl staining) to indicate the neuronal viability. Cell counting was quantified in a blinded fashion and was done in the right cerebral hemispheres located in the specific regions of dentate gyrus apex of the hippocampus. The viable cells were stained as purple‐blue and quantified with Image J software.

Moreover, the immunohistochemistry (IHC) method was utilized to evaluate the expression of TNF‐α and IL‐10 cytokines in the hippocampus of mice. Briefly, specimens fixed in buffered formalin (10%v/v) were dehydrated and embedded in paraffin. The procedure of primary antibodies and specific secondary antibodies applied in this study was according to our previous investigation (Partoazar et al., 2021) that were analyzed in a blinded fashion by an expert pathologist. The cytokines rate in the sections was interpreted semiquantitatively following scores of negative (0–1), weak (2–3), moderate (4–6), and severe (7–8). The existence of brown cells in the stained sections represented the expression of the relative tissue cytokine.

### 2.6 Statistical analysis

The data of behavioral tests and neurons number were evaluated by one‐way ANOVA followed by the parametric Tukey test and differences were expressed as mean ± SEM. The inflammatory cytokines score was analyzed with the nonparametric Kruskal–Wallis test. *p* < .05 was considered for overall significant differences among groups.

## 3 RESULTS

### 3.1 Arthrocen effect on immobility improvement

**FIGURE 1:**
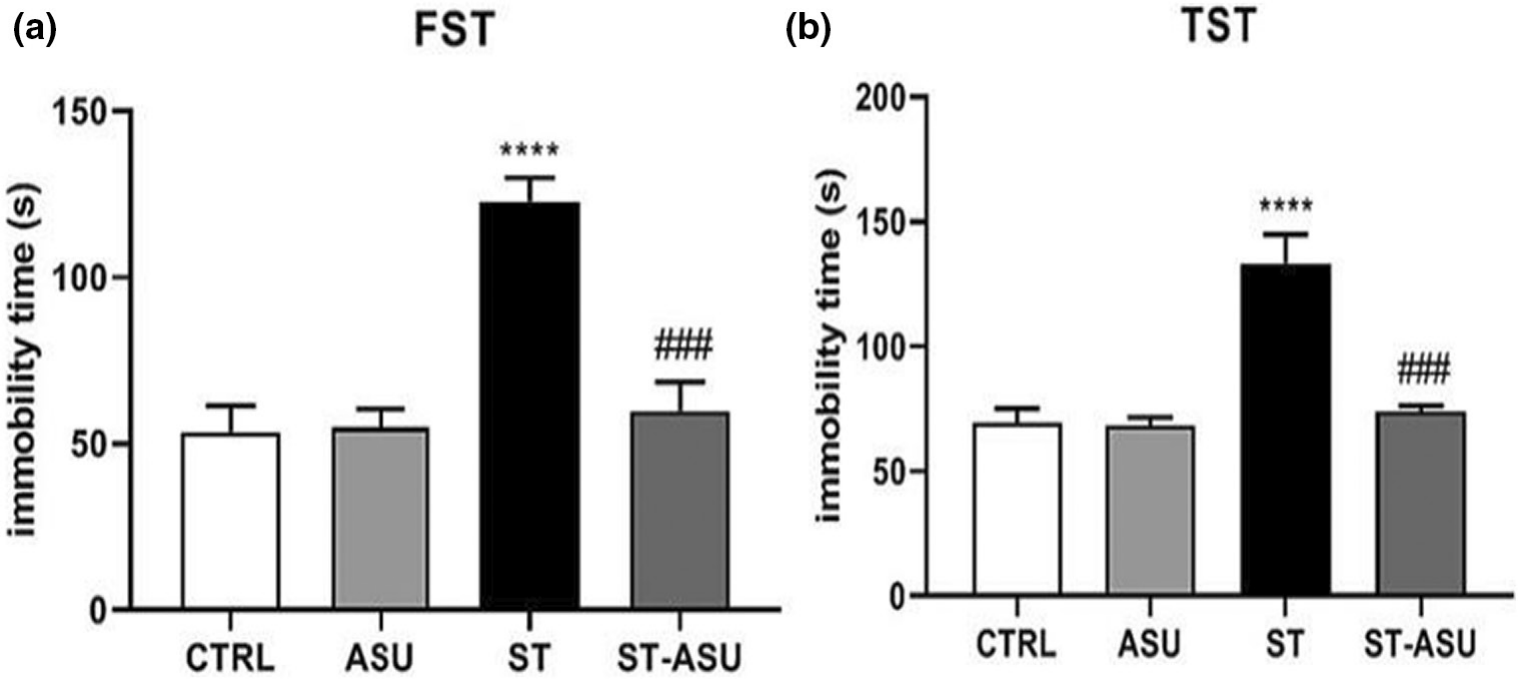
Representative data analysis of FST (a) and TST (b) methods shows that the immobility time in mice subjected to ST was reduced significantly in ASU‐treated group compared to the ST group with *n* = 6 per group and mean ± SEM. ^****^ *p* < .0001, versus CTRL group; ^###^ *p* < .001, versus ST group.

In this study, FST and TST tests were applied to investigate ASU on immobility‐like depression behavior in a PSD mice model. As results shown in Figure 1, the ST group indicated significantly increased immobility time, like depressed mood, in both relative tests in mice [(Figure 1), *p* < .0001 for FST (A) and TST (B) vs. CTRL group]. Oral ASU 50 mg/kg significantly reduced the immobility time in FST [(Figure 1a), *p* < .001, vs. ST group], and also in the TST experiment, immobility time was significantly reduced in the ST‐ASU group [(Figure 1b), *p* <.001 vs. ST group]. ASU administration without any operation on mice did not have any significant difference in behavioral activity compared to control mice.

### 3.2 Arthrocen on neuron survival

**FIGURE 2:**
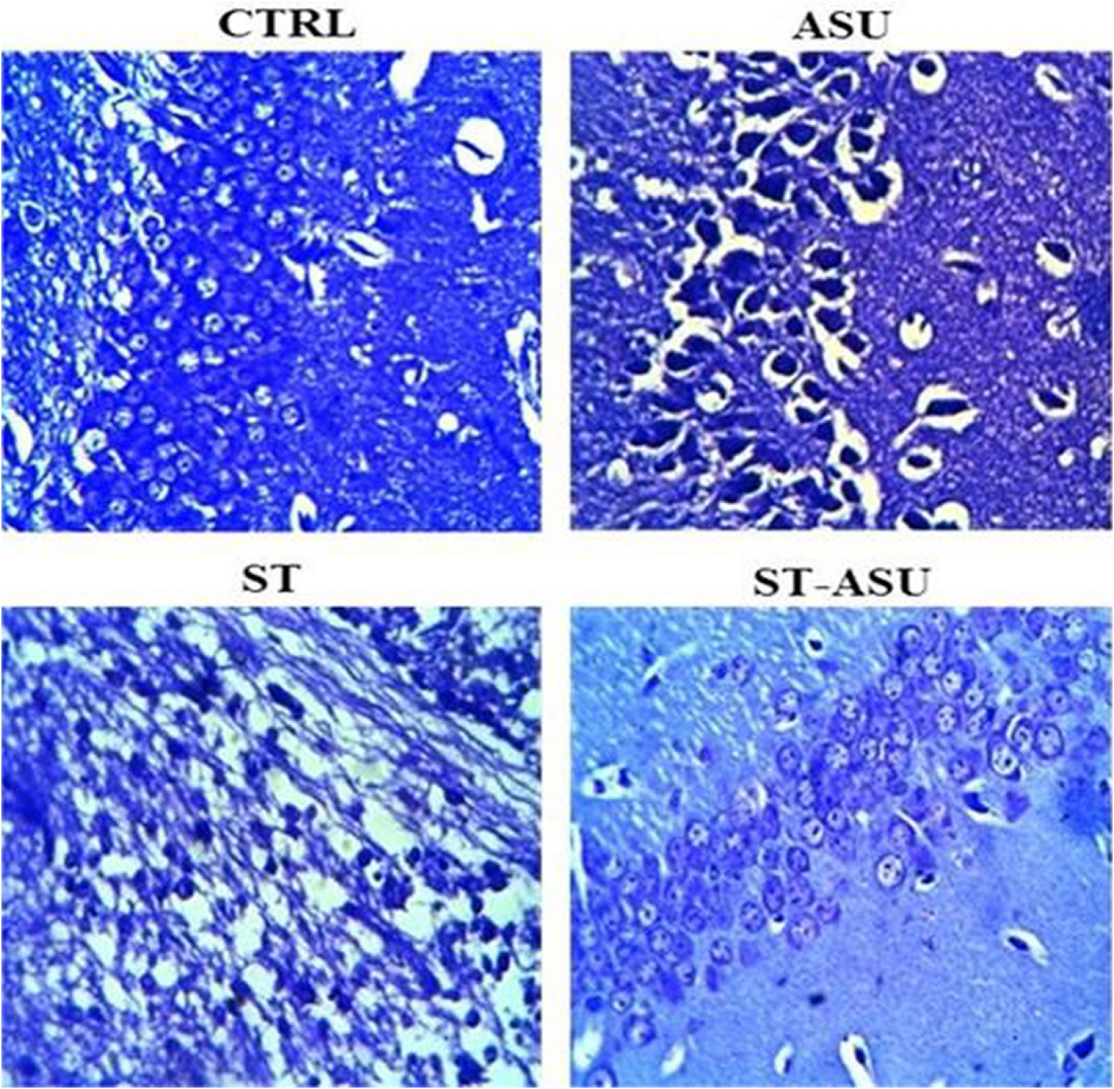
Photomicrographs of the hippocampus of the experiments with Nissl staining in 400× magnifications. Pathological lesions or neuronal damage were not seen in the CTRL group. In the ST group, the indices of decreased neuronal number and neuronal nucleus shrinkage, and cellular degeneration were observed. In the ASU‐ST group, increased healthy neurons with reduced degeneration were seen. The histomorphological pattern of ASU group’s hippocampus was similar to CTRL group with no neurological damage.

As shown in Figure 2, photomicrographs of hippocampal sections stained with Cresyl violet indicated that CTRL mice had normally shaped neurons with cytoplasmic Nissl granules in the hippocampus, whereas ST mice showed hippocampal neuronal loss and atrophy. Also, the histomorphological pattern of ASU group's hippocampus was similar to the CTRL group with no neurological damage (Figure 2). Treatment with ASU led to a significant [*p* < .0001, (Table 1)] increase in the number of viable neurons in ST mice compared to the untreated group. ANOVA analysis indicated that ST group mice had a significant lower number of viable neurons in the hippocampus compared to the CTRL group [*p* < .0001, (Table 1)].

**TABLE 1:** The number of surviving neurons was measured in the hippocampus of the different groups using Nissl staining

Experiment	CTRL	ASU	ST	ST‐ASU
Number of viable neurons	49.75 ± 1.25	49.25 ± 0.85	16.63 ± 1.66^a^	41.14 ± 4.16^b^

*Note*: Data presented are as mean ± SEM and *n* = 6.

^a^ *p* < .0001 is versus the CTRL group.

^b^ *p* < .0001 is versus the ST group.

### 3.3 Effect of Arthrocen on TNF‐α and IL‐10 levels

**FIGURE 3:**
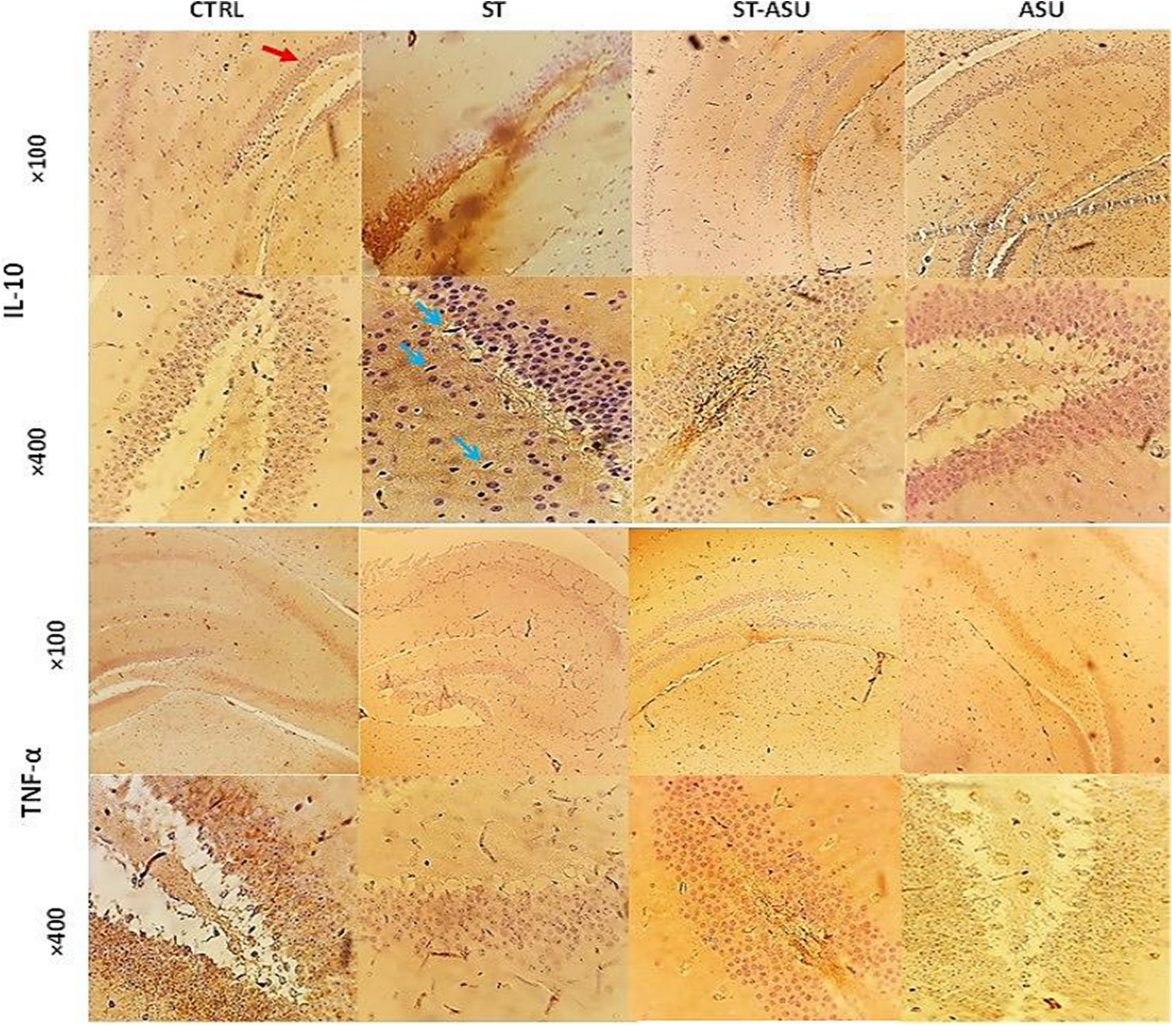
Photomicrographs of sections of the hippocampus cytokines stained with IHC in the experimental groups. The presence of brown color among the stained sections indicates the expression of the cytokine markers of TNF‐α and/or IL‐10. The red arrow at CTRL (100× & × 400) indicates the dentate gyrus (DG) region for IHC evaluation. Immune reactive cells are indicated by blue arrows in the ST group whose amounts are higher than the ST‐ASU group.

According to Figure 3, CTRL and ASU groups show that all parts of the hippocampus have normal cellularity in the dentate gyrus region in two magnifications (100× and 400×). In the ST group, TNF‐α and IL‐10 expressions increased severely [*p* < .01, (Table 2)] in comparison with control, respectively. In the ST‐ASU group compared to the ST group, IL‐10 and TNF‐α expressions were reduced significantly [*p* < .01, (Table 2)] in neural cells within a moderate level compared to ST mice. ASU administration similar to the CTRL group did not have a considerable expression of TNF‐α and IL‐10 in tissue sections (Figure 3).

**TABLE 2:** TNF‐α and IL‐10 level scoring in the hippocampal formation of mice from CTRL, ST, and ST‐ASU groups

Experiment	CTRL	ASU	ST	ST‐ASU
TNF‐α level score	0.16 ± 0.1	0.16 ± 0.1	7.5 ± 0.22^a^	4.5 ± 0.22^b^
IL‐10 level score	0.16 ± 0.1	0.16 ± 0.1	7 ± 0.12^a^	4.5 ± 0.22^b^

*Note*: Data presented are as mean ± SEM and *n* = 6.

^a^ *p* < .01 is versus the CTRL group.

^b^ *p* < .01 is versus the ST group.

## 4 DISCUSSION

The current study aimed to indicate the role of the ASU in the protection of hippocampal neurodegeneration and neuroinflammation which may be involved in depression‐like behavior in ST mice. In our experiments, the ASU‐ST group showed a significant improvement in both immobility time tests in mice subjected to 72‐h brain ischemia. Also, IHC data of the hippocampus indicated the significant reduction of pro‐inflammatory cytokines and Nissl staining showed a significant increase in the number of viable neurons in treated mice. These findings suggest that the anti‐inflammatory activity of ASU may improve depressive disorder in ST animals.

Ischemia damage is followed by systemic inflammation which subsequently causes further loss of neuronal function and cellular damage (Fang et al., 2019; Helps & Sims, 2007). Symptoms of brain ischemia damage in a large number of patients can be as depression, anxiety, and physical disabilities (Hu et al., 2021; Whyte & Mulsant, 2002). Neuropsychological deficits like depression appear as a common psychiatric consequence, with a prevalence of up to 30%–40% in patients with ST (Bartoli et al., 2013).

The experimental behavioral tests, FST and TST, are used extensively to evaluate PSD and anxiety states in rodents and to screen for effective drugs (Chen et al., 2020). It is concerned that a long duration or permanent ischemia can affect levels of immobility behavior as reliable spontaneously PSD induction in the animal models (Dandekar et al., 2022; Vahid‐Ansari et al., 2016). In our study, both behavioral tests of FST and TST confirm significant immobility in ST mice in comparison with the intact group that simulates depressive‐like behavior associated with ST (Tao et al., 2019). Besides, histopathology and IHC analysis indicated the relative outcomes containing decreased neural cells, and increased inflammatory cytokines of TNF‐α and IL‐10, respectively, in mice's ischemic brain.

The TNF‐α level usually elevates in neuroinflammatory injuries, including Alzheimer's disease (Saffari et al., 2020), encephalopathy (Zamanian et al., 2020), and ST (Bahramizadeh et al., 2019). Evidence reports referred that pro‐inflammatory cytokines imbalance like TNF‐α has a key role in the development and severity of somatic symptoms of PSD (Audet & Anisman, 2013; Zou & Crews, 2005). It has been confirmed that using anti‐TNF‐α blockers can be a probable way to help depression in unresponsive patients with PSD (Karson et al., 2013; Ma et al., 2016), where common therapies like SSRIs have shown higher levels of TNF‐α in before and posttreatment (Ma et al., 2016).

It is determined that neuronal cell damage can lead to the loss of functional activities depending on the area of injury and the amount of cell damage following cerebral ischemia (Helps & Sims, 2007). Beneficial dietary supplementation with ASU has been observed in the reduction of the seizure (Goudarzi, Zamanian, et al., 2020), and neuroprotective effect on rat hippocampus subjected to global brain ischemia (Yaman et al., 2007) in animal models.

Furthermore, our data showed that ASU consumption decreased the peak of TNF‐α cytokine level in the hippocampus of ST mice which is a typical marker of the inflammatory response. Current outcomes by ASU may result in its strong ability to suppress inflammation progression through downregulation of cytokine production in the hippocampus after cerebral ischemia (Pettigrew et al., 2016).

In the previous studies, it has been indicated that ASU administration inhibits potentially inflammatory cytokines, such as TNF‐α, IL‐1, IL‐6, IL‐8, and PGE2 via modulation of NF‐kappa B in vitro and in vivo (Au et al., 2007; Goudarzi, Partoazar, et al., 2020; Taylor et al., 2017). ASU also has the anticonvulsant effect by enhancing GABAergic neurotransmission in the mice brain (Goudarzi, Zamanian, et al., 2020).

Notably, in line with the findings of De et al. (2002) on inflamed rat microglial, our data indicated that ASU reduced IL‐10 of ST tissue brain while this cytokine increased in untreated ST. It is proposed that IL‐10 is an anti‐inflammatory, immunomodulatory cytokine that is shown to improve neurological injuries like ST (Huang, 2020) and depression (Audet & Anisman, 2013) and acts against excitotoxicity through the inhibition of TNF‐α synthesis (Bethea et al., 1999). Therefore, tempting to the controversy that retaining the ability to synthesize IL‐10 could promote neuroprotection.

Reports have shown that bioactive natural agents effectively alleviate depressive‐like immobility through their anti‐inflammatory, anti‐oxidative, and neuroprotective effects in the experimental models (Arabi et al., 2021; Hossen et al., 2021; Hua et al., 2019). It has been indicated that inflammatory factors such as TNF‐α are associated with the progression of PSD (Drieu et al., 2018; Fang et al., 2019) that could be attenuated by active agents (Hua et al., 2019; Lorigooini et al., 2021), resulting in suppression of immune response.

In conclusion, anti‐inflammatory ASU as oral consumption of 50 mg/kg/d improved significantly depression behavior associated with neuroinflammation in the hippocampus of ST mice. These outcomes suggest that depression in ST patients may be healed using supplementary ASU that warrants further investigation.

## CONFLICT OF INTEREST

All authors declare that they have no conflict of interest.

## Data Availability

Research data are not shared.
